# A shaking experience: My exposure to hypothermia

**DOI:** 10.1113/EP092839

**Published:** 2025-05-11

**Authors:** Ryan P. Sixtus

**Affiliations:** ^1^ School of Biomedical Sciences, Sir Martin Evans Building Cardiff University Glamorgan UK

At the heart of Aotearoa/New Zealand's North Island lie a range of mountains named the Tararua Range. Extending 100 km across the lower north island, these mountains are known for their steep valleys, ‘goblin forests’ and bad weather (on average the Tararua are in cloud over 280 days a year, amounting to ∼5000 mm rainfall per year). Indeed, their geography supports their reputation as ‘the traditional home of rain and fog’ (Spearpoint, 1985). Despite their short stature, ranging around 1500 m, these mountains are also notorious for receiving the howling winds that channel through the Cook Strait, frequently bringing with them freezing temperatures and sudden heavy snowfall. Like many mountains, the Tararua are a storied range, with many a hiker caught out by the ferocity of these storms – the majority by drowning in deluges flowing from these mountains, with hypothermia coming a significant second (Barnett, 2005). In a recent review of risks within the Park, the New Zealand Mountain Safety Council highlighted five top causes of incidents requiring Land Search and Rescue (LandSAR) involvement. Two of the pertinent factors for my lived experience included ‘took longer than expected’ (accounting for 38% of LandSAR call‐outs) and ‘an unexpected night out’ (accounting for 20% of LandSAR call‐outs; Walton et al., 2020). Frequently, the former was associated with onset of hypothermia and severe exhaustion, often compounding the second when people were unprepared for such a situation (Walton et al., 2020). This contributed to four of the five deaths within the park between 2010 and 2017 (Walton et al., 2020).

It was following one such midwinter storm, which brought the freezing temperatures and snow up from Antarctica, that I found myself traversing the longest continuous range in the Tararua. My proposed route was to walk from Putara, on the northern end of the Tararua Forest Park, and navigate along the Dundas Ridge to Mount Holdsworth on the Southern aspect of the park where I would rejoin the track (Figures 1 and 2).

**FIGURE 1:**
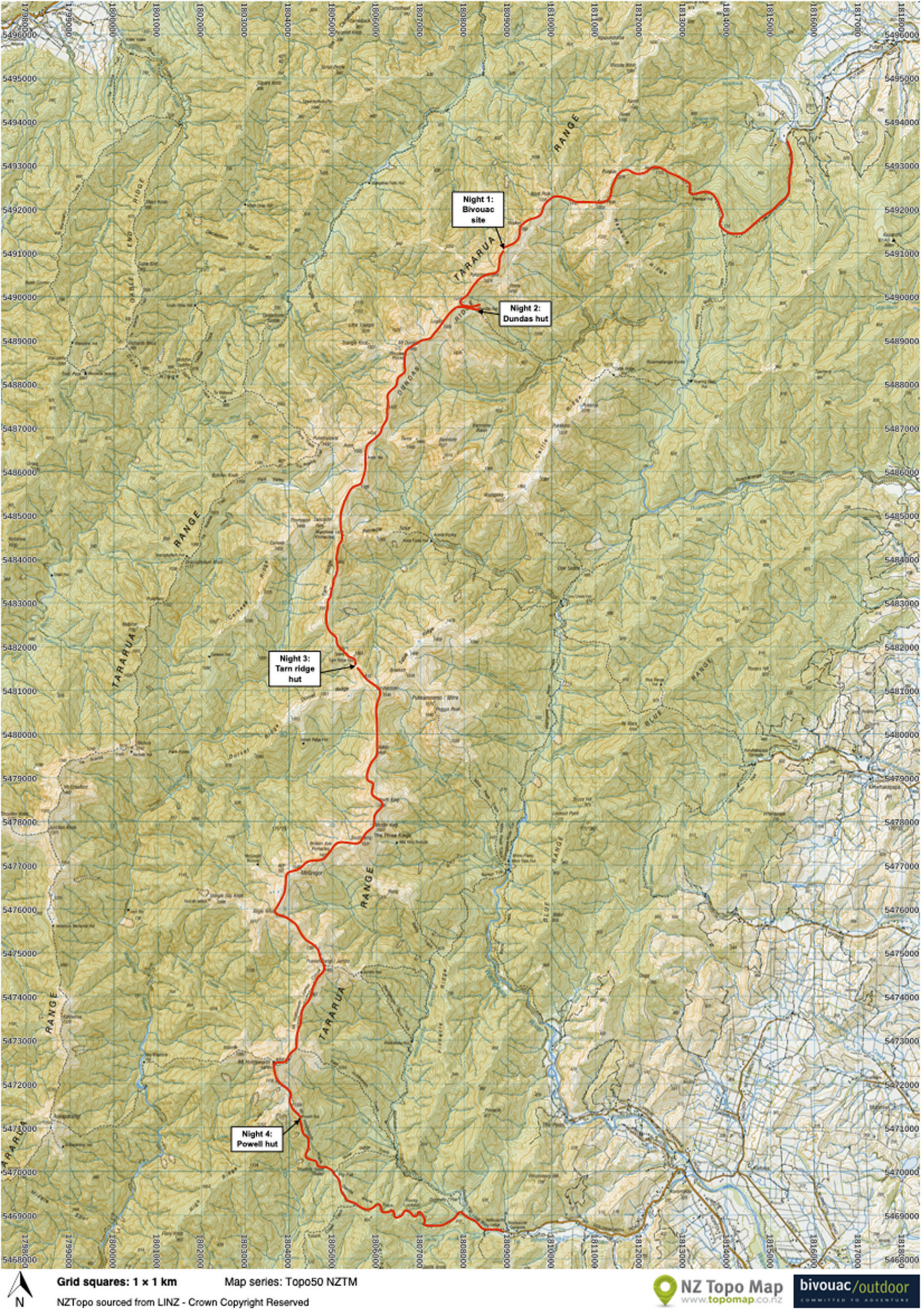
Proposed route across the Tararua range. Topographic map, curtesy of NZ Topo Map.

**FIGURE 2:**
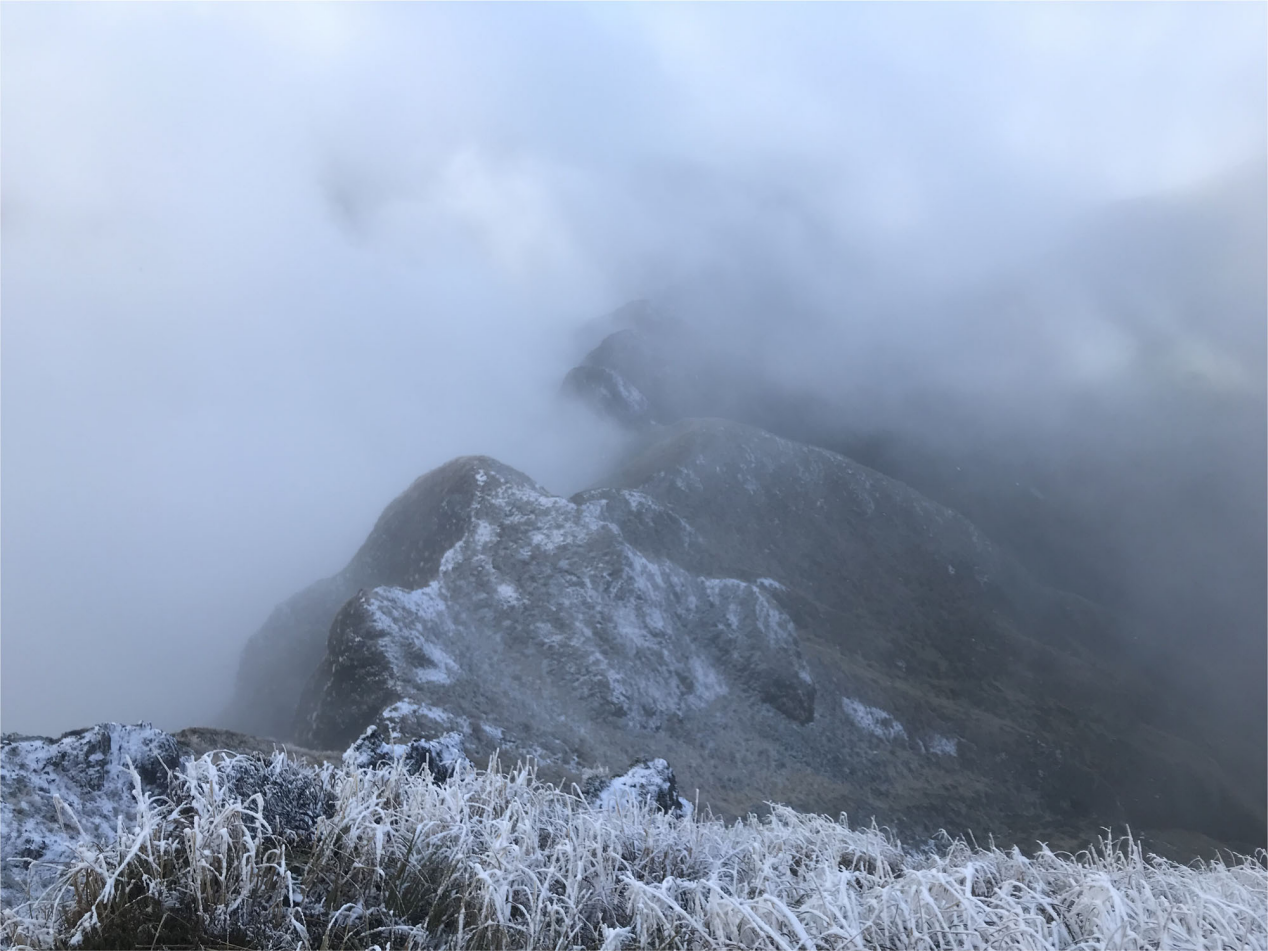
Looking across the route to Tarn Ridge hut from Arete peak (authors photograph).

The initial ascent to the alpine zone was approximately 1000 m across about 6 km. While sunny and hot at bush level (300 m), the combination of altitude and windchill makes for much colder conditions on the tops (the old rule of thumb is that temperature drops by 1°C per 100 m – actually 0.65–1°C 100 m^−1^ depending on air pressure; Brombacher, 1944). As I reached the tops, the weather began to roll in once more, and I found myself in deteriorating conditions – first with rapidly building clouds, followed closely by rain and then sleet. This first night was to be spent in Dundas hut, over the Pukemoremore mountain, however with navigation made difficult by the conditions, I found myself quickly overtaken by dusk. Due to the ascent and the load of my backpack (∼20 kg), my work rate had been high, and as such my layers relatively few – trousers, merino singlet, long‐sleeve thermal base‐layer, a mid‐layer, and shell as I reached the tops. These factors necessarily increase the physiological strain and metabolic demand, consequently reducing the physiological capacity (Fattorini et al., 2012; Phillips et al., 2016; Taylor et al., 2016). Adding wet clothes from sweating and weather exacerbates the heat loss. At 5°C, wet clothes have been shown to double heat loss compared with dry conditions (Castellani et al., 2006). As I slowed to navigate a saddle and succeeding ridgeline the weather began to cool me. Wind speeds during June 2019 averaged 22 km h^−1^ (range: 4–43 km h^−1^), with max gusts at 58 km h^−1^ (Weather & Climate, 2019). Windchill can significantly affect the ‘feel’ of the temperature, and under these conditions (0°C, wind: 22 km h^−1^) windchill can be estimated at 4184 kJ m^2^ h^−1^ (Steadman, 1971). By the time I made the decision to camp out for the night, I was likely significantly chilled. The exposed tops brought no relief, and as I began to set up camp, I was unable to retain the body heat my activity had provided, despite adding insulating layers. First, my limbs became fumbling and numb as I put the tent up, slowing my progress such that I cooled further and began to feel extremely drowsy. It was now ∼6 p.m. and conditions were worsening, with snow interspersed with dampening sleet and rain.

I set up the tent, climbed inside and immediately got into my sleeping bag. With onset of hypothermia however, external insulation – like getting in a sleeping bag – can be slow to build warmth in hypothermic individuals. This is because the thermophysiological response withdrawing blood flow from the skin layer insulates and preserves core temperature but limits conductive rewarming (Castellani & Young, 2016). In parties, body‐to‐body rewarming within a sleeping bag is often recommended (Avellanas Chavala et al., 2019), but is limited by the same mechanism. When alone, as I found myself, ensuring a hearty meal is eaten is the best policy (Paal et al., 2016). I quickly boiled a tea, followed thereafter by dinner. Consuming food has the double effect of rewarming from the core outward, as well as providing the energy needed for shivering and non‐shivering thermogenesis (Avellanas Chavala et al., 2019). Shivering thermogenesis alone can increase the metabolic rate by 5–6 times above resting levels (producing ∼720–900 kJ h^−1^ in resting exposure to cold air, and often exceeding 1259 kJ h^−1^ in cold water immersion; Castellani & Young, 2016) and may be limited by central hypoglycaemia, so consumption of energy dense foods is critical to its maintenance (Castellani & Young, 2016; Gale et al., 1981; Stocks et al., 2004). While I do not recall shivering as I cooled, certainly ceasing the effort of walking to set up my camp increased my heat loss and coupled with exhaustion, further impaired my state. Upon rewarming, I had violent – almost convulsive – shivering fits over the following 1–2 h. Having assured myself that the risk had passed, I went to sleep around 9 p.m.

The following day, the worst of the storm had abated. Exhausted and cold, I packed up my tent and moved on to Dundas hut. Reaching the hut I was almost delirious with exhaustion. I made a cup of tea, hung out my wet gear and promptly fell asleep. The energy toll in recovering from even mild hypothermia is exacting. Given that shivering can increase the metabolic rate 5–6 times above resting, and that this needs to be sustained at a maximal rate for a prolonged period of time, the cumulative toll on an individual may be as much as 2090 kJ h^−1^ (Castellani & Young, 2016). While I cannot be certain of the temperature my core reached, Stage 1 hypothermia ranges from 35°C to 32°C, and is characterised by impaired consciousness and excessive shivering (Avellanas Chavala et al., 2019; Paal et al., 2016). This level of hypothermia is insidious, and signs easily missed while hiking, as sleepiness from hypothermia can readily present as exhaustion from long hours of walking. Indeed, the latter can facilitate the former.

Upon waking, I decided to remain the night to recover fully. The subsequent section of the Tararua is both navigationally as well as technically challenging. However, I did not encounter further difficulties as I passed over Arete Peak to Tarn hut. It was within this location that a man had been reported missing and would later be found dead (Fitzgibbon, 2021: Coroner's findings). Similar conditions had driven him to seek shelter lower on the mountains and he appeared to have followed a stream that led into a ravine, common in these mountains. The coroner reported that upon entering this ravine, the man would have few choices but to forge ahead. This ultimately resulted in a fatal fall, with his body found by LandSAR after one week of searching (Fitzgibbon, 2021). During the storm this man encountered, snow on the peaks was reported to reach 1 m deep. I was fortunate in that the storm I encountered remained mostly sleet, allowing me to easily pitch a tent and move on the following day. Regardless, I still received frost nip in one of my toes, a profound exhaustion, and a shaken confidence following my brief experience with hypothermia.

Frequently in New Zealand, we read reports of a hiker going missing to later be found dead from hypothermia. Our mountains, while not excessively high nor remote, are perhaps even more dangerous for being accessible. The weather is dominated by weather patterns of Antarctica and the Pacific and are therefore highly, and rapidly, changeable. The hikers that are found dead from hypothermia are often reported as being ‘poorly prepared for the conditions’, and we local hikers shake our heads and affirm *that* will never happen to us. The reality is that with rapid changes in altitude and weather, even the most prepared tramper can be caught unprepared. Frequently, it is being cognisant of when to stop moving forward and to ‘wait out’ the storm that saves a person in the mountains. This is certainly what saved me.

## AUTHOR CONTRIBUTIONS

Sole author.

## CONFLICT OF INTEREST

The author declares that he has no competing interests.

## FUNDING INFORMATION

R.P.S. is supported by a BBSRC project grant (BB/V014765/1).
